# MRD assessed by *WT1* and *NPM1* transcript levels identifies distinct outcomes in AML patients and is influenced by gemtuzumab ozogamicin

**DOI:** 10.18632/oncotarget.2196

**Published:** 2014-07-09

**Authors:** Juliette Lambert, Jérôme Lambert, Olivier Nibourel, Cécile Pautas, Sandrine Hayette, Jean-Michel Cayuela, Christine Terré, Philippe Rousselot, Hervé Dombret, Sylvie Chevret, Claude Preudhomme, Sylvie Castaigne, Aline Renneville

**Affiliations:** ^1^ Department of Hematology, Hôpital de Versailles, Le Chesnay, Université de Versailles-Saint Quentin; France; ^2^ INSERM UMR-S 717, Paris; France; ^3^ Laboratory of Hematology, CHRU de Lille; Université de Lille Nord de France, Inserm, U837, Team 3, Cancer Research Institute of Lille, Lille; France; ^4^ Department of Hematology, Hôpital Henri Mondor, AP-HP, Créteil; France; ^5^ Laboratory of Hematology, Centre Hospitalier Lyon-Sud, Hospices Civils de Lyon, UMR5239, Université Claude Bernard, Lyon; France; ^6^ Laboratory of Hematology, Hôpital Saint-Louis, AP-HP, EA3518, University Paris Diderot, Paris; France; ^7^ Laboratory of Cytogenetics, Hôpital de Versailles, Le Chesnay; France; ^8^ Department of Hematology, Hôpital Saint Louis, AP-HP, Université Paris Diderot, EA 3518, Paris; France; ^9^ Department of Informatics and Biostatistics, Hôpital Saint Louis, Université Paris Diderot, INSERM S 717, Paris; France

**Keywords:** minimal residual disease, gemtuzumab ozogamicin, acute myeloid leukemia, WT1 expression, NPM1 mutation

## Abstract

We analysed the prognostic significance of minimal residual disease (MRD) level in adult patients with acute myeloid leukemia (AML) treated in the randomized gemtuzumab ozogamicin (GO) ALFA-0701 trial.

Levels of *WT1* and *NPM1* gene transcripts were assessed using cDNA-based real-time quantitative PCR in 183 patients with *WT1* overexpression and in 77 patients with *NMP1* mutation (*NPM1*mut) at diagnosis.

Positive *WT1* MRD (defined as > 0.5% in the peripheral blood) after induction and at the end of treatment were both significantly associated with a higher risk of relapse and a shorter overall survival (OS). Positive *NPM1*mut MRD (defined as > 0.1% in the bone marrow) after induction and at the end of treatment also predicted a higher risk of relapse, but did not influence OS. Interestingly, the achievement of a negative *NPM1*mut MRD was significantly more frequent in patients treated in the GO arm compared to those treated in control arm (39% *versus* 7% (p=0.006) after induction and 91% *versus* 61% (p=0.028) at the end of treatment). However, GO did not influence WT1 MRD levels.

Our study supports the prognostic significance of MRD assessed by *WT1* and *NPM1*mut transcript levels and show that *NPM1* MRD is decreased by GO treatment.

## INTRODUCTION

Minimal residual disease (MRD) is an important tool for assessment of response to therapy and disease follow-up in hematological malignancies. For instance, MRD monitoring is now used for European LeukemiaNet (ELN) therapeutic recommendations in patients with chronic myeloid leukemia [1]. MRD assessment is also becoming a routine procedure for treatment stratification in patients with acute lymphoblastic leukemia [2]. In AML patients, many studies have highlighted the prognostic value of MRD detection both after chemotherapy and in the pre-/post-transplant setting [3], but the use of MRD as a decision making tool has been introduced more recently [4, 5].

Two different sensitive methods, real-time quantitative PCR (RQ-PCR) and multiparameter flow cytometry (MFC), can be used to monitor MRD in AML patients. Chimeric fusion genes, such as *PML-RARA*, *RUNX1-RUNXT1* or *CBFβ-MYH11*, are reliable markers for MRD evaluation by RQ-PCR. However these markers are present in only 20-25% of AML cases. As nucleophosmin 1 gene mutations (*NPM1*mut) is a frequent marker, present in 30% of all AML patients and in 50% of those with normal karyotype, mutation-specific RQ-PCR assays have been developed for MRD monitoring [6-10].

RQ-PCR analysis of the Wilms' Tumor 1 gene (*WT1*), which is overexpressed in 70-90% AML cases [11], represents another informative marker in patients lacking specific molecular marker.

Gemtuzumab ozogamicin (GO) is an antibody-drug conjugate targeting the CD33 antigen linked to a cytotoxic derivative of the calicheamicin family of antitumor antibiotics. In the ALFA-0701 trial, we randomly evaluated the addition of fractionated doses of GO to standard-dose induction chemotherapy in patients aged 50 to 70 years old with *de novo* AML. Although complete remission (CR) rate was not significantly different between the control arm and the GO arm, 2 years event free survival (EFS) and 2 years overall survival (OS) were significantly higher in patients treated with GO (41% versus 17% and 53% versus 42%, respectively) [12]. In a recent study, we showed that independent predictors of shorter OS in ALFA-0701 study were unfavorable karyotype and SNP-A lesion(s) in the whole cohort, and SNP-A lesion(s), DNMT3A mutations and randomization in the control arm in AML with normal karyotype [13].

Throughout this study, *NPM1*mut and *WT1* transcript levels were prospectively assessed at pre-defined time-points. Here, we report on the correlation between MRD response and patient's outcome and the effect of GO on MRD levels.

## RESULTS

### Patient characteristics

**Figure 1:**
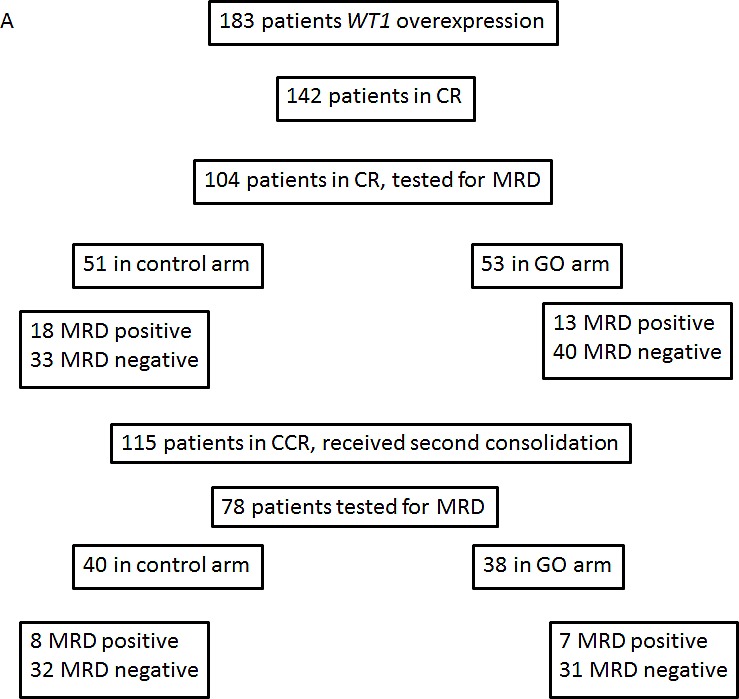

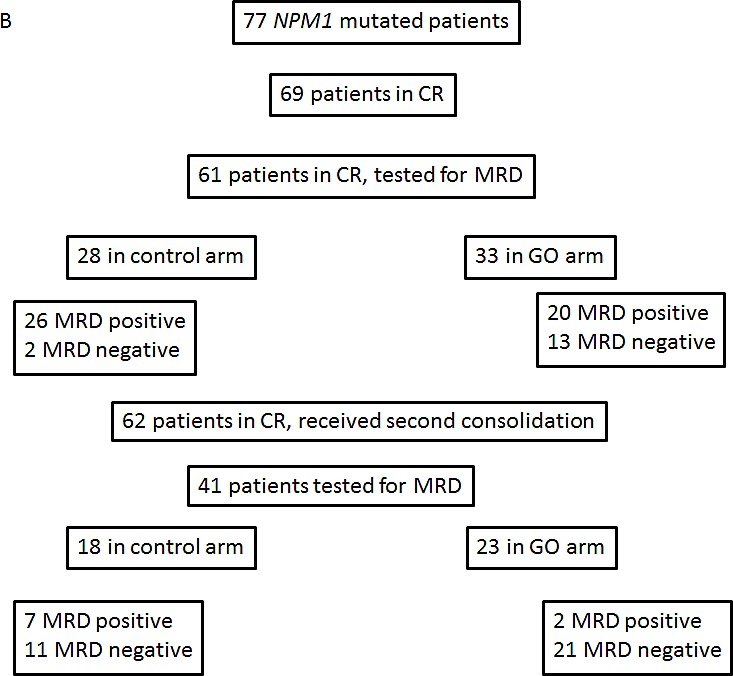
Flow chart Panel A: patients with *WT1* overexpression. Panel B: patients with *NPM1*mut.

Among the 278 patients analysed in the study, 183 (66%) had an overexpression of *WT1*, defined by a *WT1*/100*ABL* ratio at AML diagnosis above 5% in PB and/or above 25% in BM. There were 91 patients in control group and 92 patients in GO group. Main characteristics were well balanced between the two arms (Table 1). Ninety-three patients /278 (33%) had *NPM1*mut AML of which 79 had a mutation type A, B or D (42 patients in the GO arm, 37 patients in control arm). Main characteristics were well balanced, except for *FLT3*-ITD mutation which was more frequent in the control arm (Table 1). The number of MRD samples available in each treatment arm is summarized in the flow-chart (Figure 1).

**Table 1:** Characteristics of patients with *WT1* overexpression or *NMP1* mutations

*WT1*	Total (N= 183)		Control arm (N= 91)		GO arm (N= 92)		p-value
Sex (%)							0.0115
Female	101	55.2%	59	64.8%	42	45.7%	
Male	82	44.8%	32	35.2%	50	54.3%	
Median age (years) [IQR]	62.4	[58.3 - 66.4]	61.5	[57.3 - 66.0]	63	[59.6-66.8]	0.0707
WBC >50G/L	32	17.5%	13	14.3%	19	20.7%	0.331
Cytogenetics							0.318
unfavourable	44	27,0%	23	27.4%	21	26.6%	
intermediate	113	69.3%	56	66.7%	57	72.2%	
favourable	6	3.7%	5	6.0%	1	1.3%	
NA	20		7		13		
*FLT3* - ITD							0.858
negative	142	78,0%	70	76.9%	72	79.1%	
positive	40	22,0%	21	23.1%	19	20.9%	
NA	1		0		1		
*NPM1mut*							1
negative	102	56,0%	51	56,0%	51	56,0%	
positive	80	44,0%	40	44,0%	40	44,0%	
NA	1		0		1		

NPM1	Total (N= 79)		Control arm (N= 37)		GO arm (N= 42)		p-value
Sex (%)							1
Female	44	55.7%	21	56.8%	23	54.8%	
Male	35	44.3%	16	43.2%	19	45.2%	
Median age (years) [IQR]	60.6	[57.5 - 65.1]	60.1	[55.0 - 64.8]	61.4	[58.1 - 65.4]	0.306
WBC >50G/L	24	30.4%	12	32.4%	12	28.6%	0.808
Cytogenetics							1
unfavourable	2	3.1%	1	3.2%	1	2.8%	
intermediate	65	96.9%	30	96.8%	35	97.2%	
favourable	0		0		0		
NA	12		6		6		
***FLT3 - ITD***							0.0678
negative	43	55.1%	16	43.2%	27	65.9%	
positive	35	44.9%	21	56.8%	14	34.1%	
NA	1		0		1		

### Concordance in paired bone marrow and peripheral blood samples

**Figure 2:**
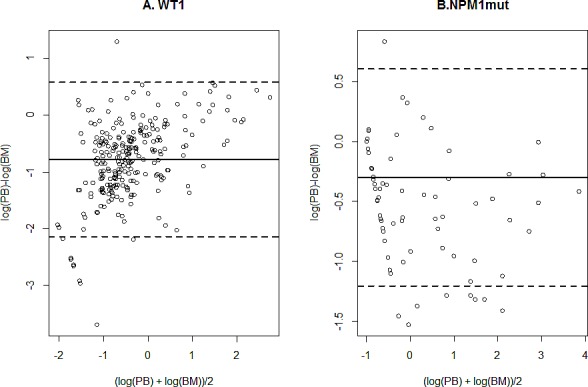
Concordance between paired PB and BM samples Bland and Altman plot of the agreement between bone marrow and peripheral blood samples measurement of *WT1* overexpression (panel A) or *NPM1* mutation (panel B) Dashed lines: limits of agreements, plain line: mean difference.

Two hundred and thirty six BM and PB paired samples of MRD were analysed in patients with *WT1* overexpression. The *WT1*/100 *ABL* ratio was - 0.71 log lower in PB as compared to BM samples (95% limits of agreement: -2.10 to 0.68) (Figure 2A). Among the 236 paired samples, 27 (11%) showed a discordant result, with a positive MRD in PB but not in BM samples in 18/27 (67%) cases. Due to this result and to the higher background level of *WT1* expression in BM samples, we decided to analyse only PB samples for MRD monitoring based on *WT1* transcript levels.

One hundred and twenty five BM and PB paired samples with *NPM1* mutation were analysed. The ratio *NPM1*mut/100 *ABL* transcripts was - 0.30 log lower in PB than in BM samples (-1.20 to 0.60) (Figure 2B). Among the 125 paired samples, 23 (18%) were discrepant with a positive MRD in BM but not in PB in 19/23 (82%) cases. We chose to analyse only BM samples for MRD monitoring based on *NPM1* mutations [14].

### Prognostic impact of MRD after induction therapy

**Figure 3:**
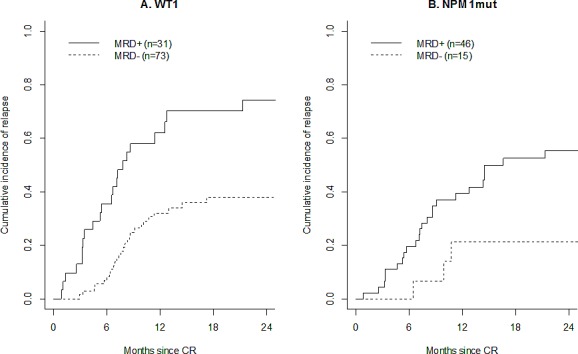
Prognostic impact of MRD after induction Cumulative incidence of relapse among patients with positive *WT1* MRD (plain line) or negative *WT1* MRD (dashed line) (panel A), and among patients with positive *NPM1*mut MRD (plain line) or negative *NPM1*mut MRD (dashed line) (panel B).

In the 104 patients who achieved a CR or CRp, a positive *WT1* MRD after induction was associated with a higher cause-specific hazard of relapse (HR=3.15 [1.78 – 5.58], p<0.0001). The cumulative incidence of relapse at 24 months was 74% (95%CI: [52 - 87%]) for 31 patients with positive MRD and 38% [26 - 50%] for 73 patients with negative MRD (Figure 3A). When adjusted for cytogenetics, randomization arm, *NPM1*mut and *FLT3*-ITD status, a positive *WT1* MRD remained independently associated with the cause-specific hazard of relapse (HR=2.45, [1.26 – 4.76], p=0.0084). These results translated to a shorter OS from CR (HR=3.23 [1.64 – 6.37], p=0.0007) in patients with a positive *WT1* MRD after induction. However, when adjusted for cytogenetics, randomization arm, *NPM1*mut and *FLT3*-ITD status, difference in OS from CR was no longer significant (HR=1.86 [0.84 – 4.12]).

Among the 61 *NPM1*mut patients who achieved a CR or CRp, a positive *NPM1*mut MRD after induction was associated with an increased cause-specific hazard of relapse: HR=3.66 [1.10 – 12.15] (p=0.035). Thus, 24-month CIR was 55% [39 – 69%] in the 46 patients with positive MRD *versus* 21% [5 – 45%] in the 15 patients with negative MRD (Figure 3B). After adjustment for treatment arm and *FLT3*-ITD status, the effect of positive *NPM1*mut MRD appeared to be similar (HR: 3.42 [0.98 – 11.96]) although no longer statistically significant. Regarding OS from CR, *NPM1*mut MRD after induction had no significant impact (HR=3.06 [0.71 – 13.24]).

### Prognostic impact of MRD at the end of treatment

**Figure 4:**
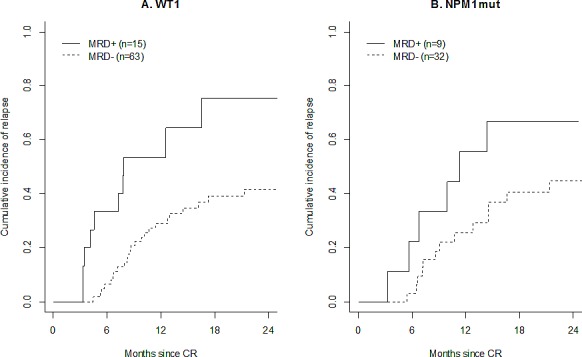
Prognostic impact of MRD at the end of treatment Cumulative incidence of relapse among patients with positive *WT1* MRD (dashed line) or negative *WT1* MRD (dashed line) (panel A), and among patients with positive *NPM1*mut MRD (plain line) or negative *NPM1*mut MRD (dashed line) (panel B).

At the end of treatment, a positive *WT1* MRD was associated with an increased cause-specific hazard of relapse (HR=3.41 [1.62 – 7.17], p=0.001). Thus, 24-month CIR was 76% [31 - 94%] in the 15 patients with positive MRD and 42% [28 - 54%] in the 63 patients with negative MRD (Figure 4A). This difference remained significant in multivariate analysis (HR=2.84 [1.05 – 7.67], p= 0.039). A positive *WT1* MRD after consolidation therapy was also associated with a shorter OS from CR (HR=6.92 [2.81 – 17.07], p<0.0001) in univariate analysis and in multivariate analysis (HR=4.64 [1.38 – 15.62], p=0.013)

Similarly, we found that *NPM1*mut MRD at the end of treatment was associated with an increased cause-specific hazard of relapse: HR=3.16 [1.18 – 8.45], p=0.022 and this association persisted after adjustment for treatment arm (HR=2.74 [1.00 – 7.54, p=0.050]). CIR at 24 months was 67% in the 9 patients with positive MRD and 45% in the 32 patients with negative MRD (Figure 4B). However, *NPM1*mut MRD response at the end of treatment did not influenced OS from CR in the cohort (HR=2.37 [0.69 – 8.19]).

### Effect of GO treatment on MRD

**Figure 5:**
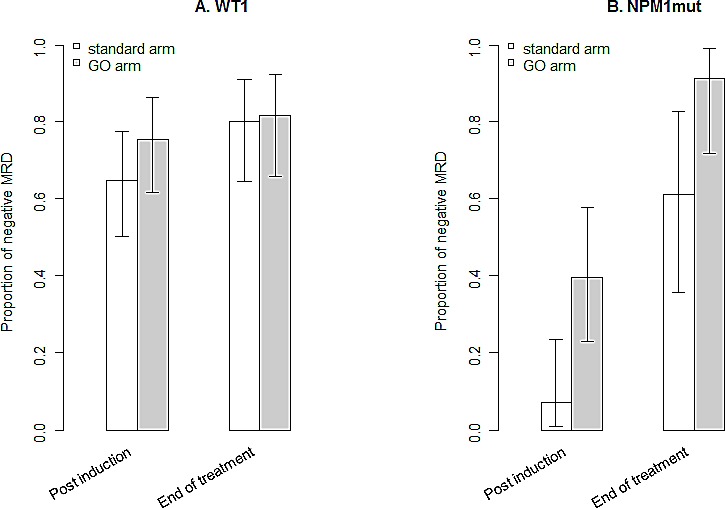
Effect of GO treatment on MRD Proportion of patients with a negative MRD according to treatment arm, *WT1* MRD (panel A) and *NPM1*mut MRD (panel B).

GO treatment had no effect on *WT1* MRD negativity, either after induction therapy (75% of *WT1* MRD negative patient in the GO arm *versus* 65% in the control arm, p=0.29) or at the end of treatment (82% *versus* 80%, p=1). When the analysis was restricted to the subset of patients with favourable or intermediate karyotype, results were similar (Figure 5A).

A negative *NPM1*mut MRD was more frequently observed in patients treated in the GO arm compared to those treated in the control arm: 39% *versus* 7% (p=0.006) achieved MRD negativity after induction therapy and 91% *versus* 61% (p=0.028) at the end of treatment, respectively in the GO arm and in the control arm (Figure 5B). When adjusted for *FLT3*-ITD status, treatment with GO was still independently associated with *NPM1*mut** MRD negativity both after induction and at the end of treatment.

## DISCUSSION

Here, we report correlation between MRD levels and patient outcome, using sensitive cDNA – based RQ-PCR assays of two molecular MRD markers, *WT1* overexpression and *NPM1* mutations in a homogeneous population of AML patients aged 50 to 70 years treated in the multicentric randomized ALFA-0701 trial, which tested the addition of GO to conventional chemotherapy.

With these two markers, 190 patients /278 (68%) enrolled in this study were informative at diagnosis for MRD monitoring. MRD data were available in 115 patients /149 (77%) informative patients in CR or CRp after induction and in 86 patients /122 (70%) in continuous response at the end of treatment. Seventy two patients were positive for both markers.

Our results showed that *WT1* MRD was associated with an increase cause specific hazard of relapse and with a shorter OS from CR. These results remained significant in multivariate analysis. Regarding *NPM1*mut patients, a positive MRD was associated with an increase cause specific hazard of relapse. Correlation between *NPM1*mut MRD and survival duration was not observed, probably because of the smaller number of patients but we cannot excluded others explanations, as selection of a resistant clone [15] or impact of *DNMT3A* mutation, which is frequently associated with *NPM1* mutations[16].

The prognostic value of *NPM1*mut MRD on patient's outcome had been previously reported in younger adults with AML [7-9]. We observed the same results in a population of older patients. The value of MRD monitoring based on *WT1* transcript levels has been debated because of the difficulty to discriminate the residual expression of leukemic cells from the background expression. However, several studies have shown a correlation between a detectable *WT1* MRD and clinical outcomes [17-21]. The validity of *WT1* transcript levels was acknowledged in the ELN consortium guidelines with a standardized method and the authors proposed threshold for MRD detection in BM and PB samples [11]. In our study, concordance analysis between paired BM and PB samples showed that *WT1* MRD levels were more frequently positive in PB than in the corresponding BM samples and we chose to analyse only PB samples. Our results eventually showed that *WT1* MRD level measured in PB samples was well correlated with relapse risk and OS from CR, although *WT1* is a less sensible marker than *NPM1*mut.

In this study, we compared MRD levels in patients treated in the control arm and in the GO arm. The proportion of patients with a negative *NPM1*mut MRD was significantly superior after induction and at the end of treatment in the GO arm as compared to control arm. Correlation between MRD negativity and GO treatment was not observed with *WT1* transcript levels probably because of the lower sensitivity of this marker for which the median MRD reduction between diagnosis and remission samples was only 1 log. In the ALFA-0701 study as in the AML16 MRC study, GO treatment was associated with a benefit in survival endpoints although there was no difference in CR rates [12, 22]. It has been shown that assessment of treatment response is more informative by MRD monitoring than by conventional morphology [23, 24]. Our results in *NPM1*mut patients argued in favour of a better quality of remission in patients treated with GO, which can be related to the better outcome observed with GO treatment.

Two recent studies have reported results of MRD monitoring by MFC in patients treated with GO. In a non-randomized pediatric study, 232 AML children received chemotherapy or GO plus chemotherapy or GO alone according to MRD levels assessed by MFC after first induction. GO alone or in combination with chemotherapy reduced MRD levels: 13/17 MRD positive patients treated with GO alone and 13/29 MRD positive patients treated with GO plus chemotherapy became MRD negative [25]. In the randomized MRC AML16 trial, MRD negativity, assessed by MFC, after induction was correlated with OS in the whole patient cohort. However, treatment with GO was not associated with MRD negativity [5]. Thus, our randomized study demonstrated for the first time a direct impact of GO treatment on MRD assessed by *NPM1*mut levels in adult AML patients, which could be related to the higher cumulative dose of GO administered during induction in ALFA-0701 study.

Overall, our results confirm the prognostic significance of MRD based on *WT1* overexpression and *NPM1* mutations in AML. Interestingly, we showed that treatment with GO significantly improved molecular response assessed by *NPM1*mut detection after induction therapy and at the end of treatment. Finally, our results suggest that MRD assessment after induction may be used as a surrogate marker in AML, of interest in the context of news drugs development.

## METHODS

### Patients and treatment

Two hundred and seventy eight patients aged 50 to 70 years old, with previously untreated *de novo* AML were enrolled in the ALFA-0701 trial. Patients were randomly assigned to receive standard induction chemotherapy with daunorubicin 60 mg/m^2^ at day 1, 2 and 3 and cytarabine 200 mg/m^2^ in continuous infusion from D1 to D7 with or without GO in fractioned 3 mg/m^2^ doses (maximum: 5 mg/dose) at D1, D4 and D7. Then, patients in CR received two consolidation courses with cytarabine and daunorubicin with or without GO 3 mg/m^2^ (maximum: 5 mg) at D1, according to their initial randomization arm.

Collection of bone marrow (BM) and peripheral blood (PB) samples was recommended by the protocol at AML diagnosis, after induction therapy, and after each consolidation courses. All patients gave informed consent for both treatment and genetic analysis before inclusion, according to the declaration of Helsinki. The study is registered with EudraCT, number 2007-002933-36 and with ClinicalTials.gov Identifier NCT00927498.

### Cytogenetic analysis

Cytogenetic R-banding analysis was performed on diagnostic BM samples using standard methods. Karyotypes were described according to the International System for Human Cytogenetic Nomenclature recommendations[26] and classified within favorable, intermediate and unfavorable groups[12].

### Gene mutations analysis

*NPM1*mut screening was performed by PCR and fragment analysis as previously described [27]. Samples showing a mutated profile were then analyzed by direct Sanger sequencing in order to identify the type of mutation. *NPM1* exon 12 was amplified by PCR from genomic DNA using the HotStar Taq DNA Polymerase Kit (Qiagen, Courtaboeuf, France). Purified PCR products were subsequently sequenced twice in reverse direction using the BigDye Terminator Cycle Sequencing Kit (Applied Biosystems, Courtaboeuf, France) and analyzed on the Applied Biosystems 3130xl Genetic Analyzer. Data were analyzed with the SeqScape software version 2.5. *FLT3* internal tandem duplications (*FLT3*-ITD) were screened as previously described [28].

### Quantification of *NPM1*mut transcript levels

The assessment of *NPM1*mut transcript levels was performed on an ABI Prism 7900 platform (Applied Biosystems) with a mutation-specific RQ-PCR assay. For *NPM1* mutation type B, we applied the RQ-PCR assay designed by Gorello et al [6]. For *NPM1* mutations types A and D, we used primers and probe and PCR conditions described by Krönke et al [9] to minimize the wild-type background. Plasmids from Ipsogen (Marseille, France) were used to establish the standard curves. MRD levels were reported as the normalized values of *NPM1*mut copy number/*ABL* copy number x 100 (%). The quantitative detection limit of the assays was 0.1%. The achievement of MRD levels below this threshold was hereafter defined as a negative MRD.

### Quantification of *WT1* expression levels

The quantification of *WT1* transcripts was performed on an ABI Prism 7900 platform using the standardized ELN RQ-PCR assay. *WT1* mRNA levels were normalized to *ABL* control gene and results were expressed as the ratio *WT1* copy number / *ABL* copy number x 100 (%). The upper limit of normal was defined as 2.5% in BM samples and 0.5% in PB samples, as recommended in the ELN study [11]. We considered that *WT1* was overexpressed at AML diagnosis when *WT1* mRNA level was ≥ 10-fold the upper limit of normal (i.e., 25% in BM and 5% in PB samples). The achievement of MRD levels below 2.5% in BM or 0.5% in PB was hereafter defined as a negative MRD.

### Statistical analysis

Data are presented as median (25th-75th percentile) for quantitative variables and count (percentage) for qualitative variables.

First, agreement of MRD measurement between paired BM and PB samples was assessed using the Bland and Altman plot [29]. Such plots compute 95% limits of agreement for each comparison (average difference ± 1.96 standard deviation of the difference), which tell us how far apart measurements by the two methods are more likely to be for most individuals, allowing to investigate the existence of any systematic difference between the measurements (i.e., fixed bias) and to identify possible outliers. Otherwise, we computed the percentage of discordant results (i.e., MRD positive in one sample and negative in the other) using traditional cut-offs, namely 2% in BM samples and 0.5% in PB samples for *WT1* overexpression, and 0.1% in BM and PB samples for *NPM1*mut.

The evaluation of the predictive value on the occurrence of death or relapse, of the MRD levels after induction or after consolidation according to previous thresholds, was restricted to the subsample of patients in complete remission. The effect of MRD levels on OS was assessed through a proportional hazard Cox model. When considering the effect of MRD levels on the occurrence of relapse, and to account for the competing events of death without relapse and hematopoietic stem cell transplantation, we modeled the cause-specific hazard of relapse using Cox models. Results were adjusted for treatment arm, cytogenetic and *FLT3*-ITD status. At last, cumulative incidences of relapse (CIR) were plotted.

Finally, the effect of GO on MRD negativity was assessed by a Fisher's exact test comparing negativity of MRD after induction and at the end of treatment across the treatment arms. This comparison was then adjusted for *FLT3*-ITD status.

Statistical analyses were performed on R (http://www.R-project.org/) software. All p-values were two-sided, with p<0.05 denoting statistical significance.

## References

[1] Baccarani M, Cortes J, Pane F, Niederwieser D, Saglio G, Apperley J, Cervantes F, Deininger M, Gratwohl A, Guilhot F, Hochhaus A, Horowitz M, Hughes T, Kantarjian H, Larson R, Radich J (2009). Chronic myeloid leukemia: an update of concepts and management recommendations of European LeukemiaNet. J Clin Oncol.

[2] Eckert C, Henze G, Seeger K, Hagedorn N, Mann G, Panzer-Grumayer R, Peters C, Klingebiel T, Borkhardt A, Schrappe M, Schrauder A, Escherich G, Sramkova L, Niggli F, Hitzler J, von Stackelberg A (2013). Use of allogeneic hematopoietic stem-cell transplantation based on minimal residual disease response improves outcomes for children with relapsed acute lymphoblastic leukemia in the intermediate-risk group. J Clin Oncol.

[3] DiNardo CD, Luger SM (2012). Beyond morphology: minimal residual disease detection in acute myeloid leukemia. Curr Opin Hematol.

[4] Terwijn M, van Putten WL, Kelder A, van der Velden VH, Brooimans RA, Pabst T, Maertens J, Boeckx N, de Greef GE, Valk PJ, Preijers FW, Huijgens PC, Drager AM, Schanz U, Jongen-Lavrecic M, Biemond BJ (2013). High prognostic impact of flow cytometric minimal residual disease detection in acute myeloid leukemia: data from the HOVON/SAKK AML 42A study. J Clin Oncol.

[5] Freeman SD, Virgo P, Couzens S, Grimwade D, Russell N, Hills RK, Burnett AK (2013). Prognostic relevance of treatment response measured by flow cytometric residual disease detection in older patients with acute myeloid leukemia. J Clin Oncol.

[6] Gorello P, Cazzaniga G, Alberti F, Dell'Oro MG, Gottardi E, Specchia G, Roti G, Rosati R, Martelli MF, Diverio D, Lo Coco F, Biondi A, Saglio G, Mecucci C, Falini B (2006). Quantitative assessment of minimal residual disease in acute myeloid leukemia carrying nucleophosmin (NPM1) gene mutations. Leukemia.

[7] Chou WC, Tang JL, Wu SJ, Tsay W, Yao M, Huang SY, Huang KC, Chen CY, Huang CF, Tien HF (2007). Clinical implications of minimal residual disease monitoring by quantitative polymerase chain reaction in acute myeloid leukemia patients bearing nucleophosmin (NPM1) mutations. Leukemia.

[8] Schnittger S, Kern W, Tschulik C, Weiss T, Dicker F, Falini B, Haferlach C, Haferlach T (2009). Minimal residual disease levels assessed by NPM1 mutation-specific RQ-PCR provide important prognostic information in AML. Blood.

[9] Kronke J, Schlenk RF, Jensen KO, Tschurtz F, Corbacioglu A, Gaidzik VI, Paschka P, Onken S, Eiwen K, Habdank M, Spath D, Lubbert M, Wattad M, Kindler T, Salih HR, Held G (2011). Monitoring of minimal residual disease in NPM1-mutated acute myeloid leukemia: a study from the German-Austrian acute myeloid leukemia study group. J Clin Oncol.

[10] Jain P, Kantarjian H, Patel K, Faderl S, Garcia-Manero G, Benjamini O, Borthakur G, Pemmaraju N, Kadia T, Daver N, Nazha A, Luthra R, Pierce S, Cortes J, Ravandi F (2013). Mutated NPM1 in patients with acute myeloid leukemia in remission and relapse. Leuk Lymphoma.

[11] Cilloni D, Renneville A, Hermitte F, Hills RK, Daly S, Jovanovic JV, Gottardi E, Fava M, Schnittger S, Weiss T, Izzo B, Nomdedeu J, van der Heijden A, van der Reijden BA, Jansen JH, van der Velden VH (2009). Real-time quantitative polymerase chain reaction detection of minimal residual disease by standardized WT1 assay to enhance risk stratification in acute myeloid leukemia: a European LeukemiaNet study. J Clin Oncol.

[12] Castaigne S, Pautas C, Terre C, Raffoux E, Bordessoule D, Bastie JN, Legrand O, Thomas X, Turlure P, Reman O, de Revel T, Gastaud L, de Gunzburg N, Contentin N, Henry E, Marolleau JP (2012). Effect of gemtuzumab ozogamicin on survival of adult patients with de-novo acute myeloid leukaemia (ALFA-0701): a randomised, open-label, phase 3 study. Lancet.

[13] Renneville A, Abdelali RB, Chevret S, Nibourel O, Cheok M, Pautas C, Dulery R, Boyer T, Cayuela JM, Hayette S, Raffoux E, Farhat H, Boissel N, Terre C, Dombret H, Castaigne S (2014). Clinical impact of gene mutations and lesions detected by SNP-array karyotyping in acute myeloid leukemia patients in the context of gemtuzumab ozogamicin treatment: results of the ALFA-0701 trial. Oncotarget.

[14] Shayegi N, Kramer M, Bornhauser M, Schaich M, Schetelig J, Platzbecker U, Rollig C, Heiderich C, Landt O, Ehninger G, Thiede C (2013). The level of residual disease based on mutant NPM1 is an independent prognostic factor for relapse and survival in AML. Blood.

[15] Blagosklonny MV (2005). Why therapeutic response may not prolong the life of a cancer patient: selection for oncogenic resistance. Cell Cycle.

[16] LaRochelle O, Bertoli S, Vergez F, Sarry JE, Mansat-De Mas V, Dobbelstein S, Dastugue N, Strzelecki AC, Cavelier C, Creancier L, Pillon A, Kruczynski A, Demur C, Sarry A, Huguet F, Huynh A (2011). Do AML patients with DNMT3A exon 23 mutations benefit from idarubicin as compared to daunorubicin? A single center experience. Oncotarget.

[17] Lapillonne H, Renneville A, Auvrignon A, Flamant C, Blaise A, Perot C, Lai JL, Ballerini P, Mazingue F, Fasola S, Dehee A, Bellman F, Adam M, Labopin M, Douay L, Leverger G (2006). High WT1 expression after induction therapy predicts high risk of relapse and death in pediatric acute myeloid leukemia. J Clin Oncol.

[18] Lasa A, Carricondo M, Estivill C, Bussaglia E, Gich I, Brunet S, Aventin A, Sierra J, Nomdedeu JF (2009). WT1 monitoring in core binding factor AML: comparison with specific chimeric products. Leuk Res.

[19] Cilloni D, Gottardi E, Fava M, Messa F, Carturan S, Busca A, Guerrasio A, Saglio G (2003). Usefulness of quantitative assessment of the WT1 gene transcript as a marker for minimal residual disease detection. Blood.

[20] Cilloni D, Messa F, Arruga F, Defilippi I, Gottardi E, Fava M, Carturan S, Catalano R, Bracco E, Messa E, Nicoli P, Diverio D, Sanz MA, Martinelli G, Lo-Coco F, Saglio G (2008). Early prediction of treatment outcome in acute myeloid leukemia by measurement of WT1 transcript levels in peripheral blood samples collected after chemotherapy. Haematologica.

[21] Nowakowska-Kopera A, Sacha T, Florek I, Zawada M, Czekalska S, Skotnicki AB (2009). Wilms' tumor gene 1 expression analysis by real-time quantitative polymerase chain reaction for monitoring of minimal residual disease in acute leukemia. Leuk Lymphoma.

[22] Burnett AK, Russell NH, Hills RK, Kell J, Freeman S, Kjeldsen L, Hunter AE, Yin J, Craddock CF, Dufva IH, Wheatley K, Milligan D (2012). Addition of gemtuzumab ozogamicin to induction chemotherapy improves survival in older patients with acute myeloid leukemia. J Clin Oncol.

[23] Loken MR, Alonzo TA, Pardo L, Gerbing RB, Raimondi SC, Hirsch BA, Ho PA, Franklin J, Cooper TM, Gamis AS, Meshinchi S (2012). Residual disease detected by multidimensional flow cytometry signifies high relapse risk in patients with de novo acute myeloid leukemia: a report from Children's Oncology Group. Blood.

[24] Inaba H, Coustan-Smith E, Cao X, Pounds SB, Shurtleff SA, Wang KY, Raimondi SC, Onciu M, Jacobsen J, Ribeiro RC, Dahl GV, Bowman WP, Taub JW, Degar B, Leung W, Downing JR (2012). Comparative analysis of different approaches to measure treatment response in acute myeloid leukemia. J Clin Oncol.

[25] O'Hear C, Inaba H, Pounds S, Shi L, Dahl G, Bowman WP, Taub JW, Pui CH, Ribeiro RC, Coustan-Smith E, Campana D, Rubnitz JE (2013). Gemtuzumab ozogamicin can reduce minimal residual disease in patients with childhood acute myeloid leukemia. Cancer.

[26] Mitelman FE (1992). ISCN 1991: guidelines for cancer cytogenetics: supplement to an International System for Human Cytogenetic Nomenclature: Karger.

[27] Boissel N, Renneville A, Biggio V, Philippe N, Thomas X, Cayuela JM, Terre C, Tigaud I, Castaigne S, Raffoux E, De Botton S, Fenaux P, Dombret H, Preudhomme C (2005). Prevalence, clinical profile, and prognosis of NPM mutations in AML with normal karyotype. Blood.

[28] Renneville A, Boissel N, Nibourel O, Berthon C, Helevaut N, Gardin C, Cayuela JM, Hayette S, Reman O, Contentin N, Bordessoule D, Pautas C, Botton S, Revel T, Terre C, Fenaux P (2012). Prognostic significance of DNA methyltransferase 3A mutations in cytogenetically normal acute myeloid leukemia: a study by the Acute Leukemia French Association. Leukemia.

[29] Bland JM, Altman DG (1986). Statistical methods for assessing agreement between two methods of clinical measurement. Lancet.

